# Emerging threat and vaccination strategies of H9N2 viruses in poultry in Indonesia: A review

**DOI:** 10.12688/f1000research.118669.1

**Published:** 2022-05-19

**Authors:** Saifur Rehman, Fedik Abdul Rantam, Khadija Batool, Aamir Shehzad, Mustofa Helmi Effendi, Adiana Mutamsari Witaningrum, Muhammad Bilal, Muhammad Thohawi Elziyad Purnama

**Affiliations:** 1 Division of Veterinary Public Health Faculty of Veterinary Medicine, Universitas Airlangga, Surabaya, East Java, 60115, Indonesia; 2 Laboratory of Virology and Immunology Division of Microbiology, Faculty of Veterinary Medicine, Universitas Airlangga, Surabaya, East Java, 60115, Indonesia; 3 Epidemiology and Public Health, University of Veterinary and Animal Sciences, Lahore, Islamic, 40050, Pakistan; 4 Medicine, Service Institute of Medical Sciences, Lahore,, Punjab, 40050, Pakistan; 5 Division of Veterinary Anatomy, Department of Veterinary Science, Faculty of Veterinary Medicine, Universitas Airlangga, Surabaya, East Java, 60115, Indonesia

**Keywords:** avian influenza, public health, emergence, vaccination, Indonesia

## Abstract

Avian influenza virus subtype H9N2 was first documented in Indonesia in 2017. It has become prevalent in chickens in many provinces of Indonesia as a result of reassortment in live bird markets. Low pathogenic avian influenza subtype H9N2 virus-infected poultry provides a new direction for influenza virus. According to the latest research, the Indonesian H9N2 viruses may have developed through antigenic drift into new genotype, posing a significant hazard to poultry and public health. The latest proof of interspecies transmission proposes that, the next human pandemic variant will be avian influenza virus subtype H9N2. Manipulation and elimination of H9N2 viruses in Indonesia, constant surveillance of viral mutation, and vaccines updates are required to achieve effectiveness. The current review examines should be investigates/assesses/report on the development and evolution of newly identified H9N2 viruses in Indonesia and their vaccination strategy.

## Introduction

The avian influenza virus subtype H9N2 is a LPAIV widely circulated in Asian poultry. ^1^ In the future, the LPAI H9N2 virus like H5N1 could pose a serious zoonotic threat ^2^ because they have been isolated from backyard and wild bird species. It was discovered in a variety of avian species throughout Eurasia, the poultry industry has suffered significant financial losses as a result of this. ^3^ The H9N2 virus has gained much attention due to its rapid dispersion between native birds. ^4^ This low pathogenic virus survive in chicks and transmits to unaffected birds via the fecal-oral route despite causing extreme clinical signs. ^5^ The avian influenza virus subtype H9N2 causes severe respiratory illness in immunocompromised chickens. It causes an increase in early chick mortality as well as a considerable decline in egg production in laying chickens, resulting in financial loss. ^6^ When this virus is co-infected with other pathogens, the intensity of clinical symptoms, death rates, and viral replication can increase. ^7^^,^^8^ Based on their genetic and antigenic properties H9N2 viruses prevalent in Asia have been classified into three genotypes: A/Quail/Hong Kong/G1/97-like (G1-like); the Y280 lineage, represented by A/Chicken/Hong Kong/Y280/97-like (Y280-like); and the Korean lineage, represented by A/Chicken/Korea/38349-p96323/96 (Korean-like). ^9^ The G1 prototype virus (A/Quail/Hong Kong/G1/97) is common in southern Chinese quail. It may have been the source of internal genes for the highly pathogenic avian influenza (HPAI) subtype H5N1 that hit Hong Kong in 1997. H9N2 viruses with G1 lineages have been found in field epidemics of influenza in poultry in the Middle East and the Indian subcontinent since 1997. Since the early 1990s, H9N2 has evolved to create a more diversified genotype in grassland poultry birds by acquiring gene fragments from other viruses. The genomes of newly isolated avian influenza (H9N2) viruses showed significant genetic recombination in HPAI viruses. ^10^^–^^12^

**Figure 1.**
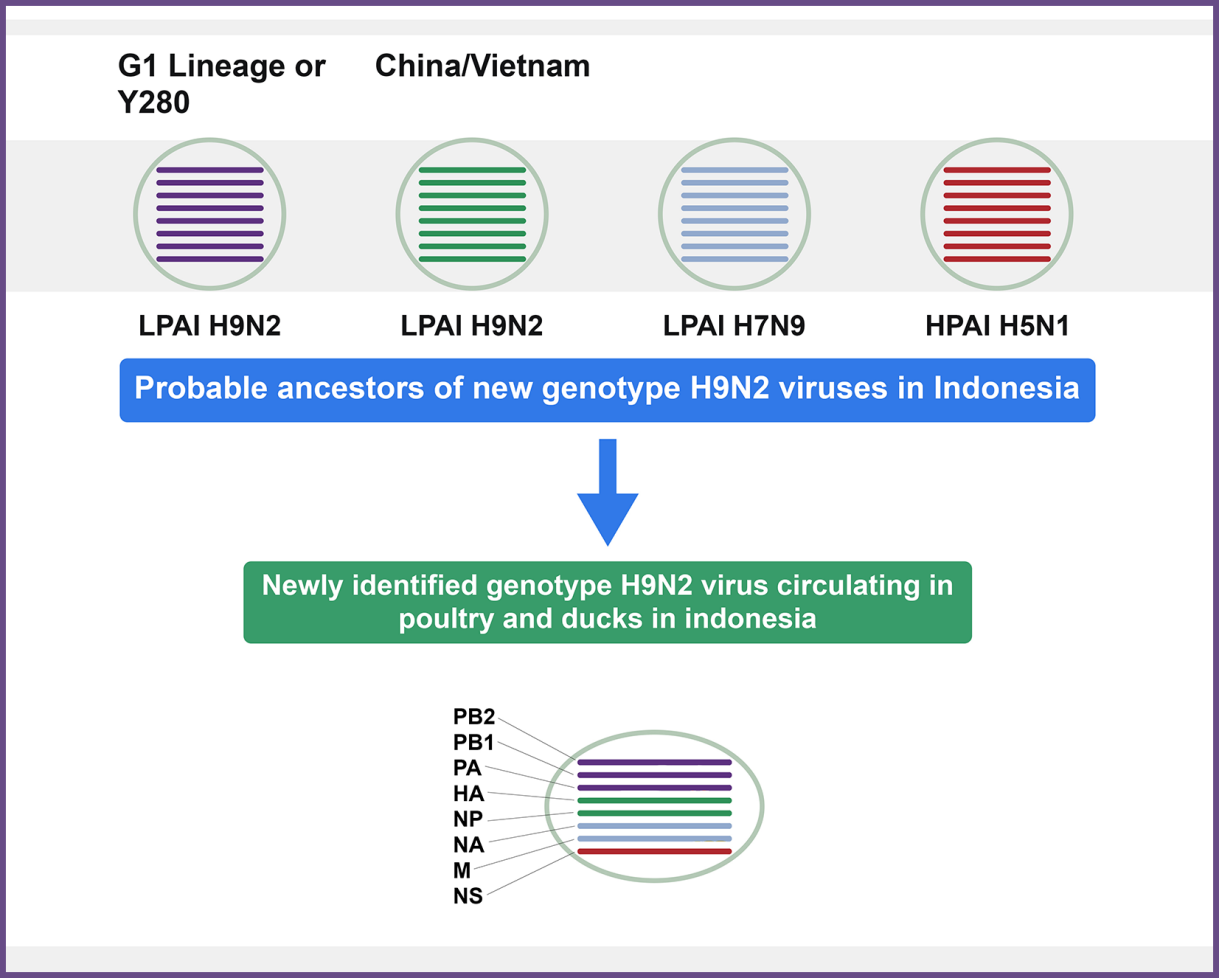
Representation of recently emerged H9N2 virus genotypes in poultry in Indonesia. ^86^

A novel H9N2 genotype, expressed by A/chicken/West Java/BBLitvet-RI/2017, A/chicken/East Java/Spg147/2018, A/chicken/East Java/BLi25Ut/2018 and A/chicken/Central Java/SLO.105/2018 was isolated from Indonesian poultry birds, and these replaced by Y280 or G1 Lineage. ^13^^,^^14^ Inter- and Intra-subtype genotype genomic recombination between LPAIV subtype H9N2 (G1-like), HPAIV subtype H5N1 (clade 2.2), and H7N9 viruses resulted in these novel reassortants ( Figure 1). A novel H9N2 genotype in Indonesia represented 98% sequence identity with that of (A/Muscovy duck/Vietnam/LBM719/2014(H9N2) was isolated from chicken in a study conducted by Melina Jonas. ^15^ Co-circulation of the LPAI virus subtype H9N2 has been reported in Egypt with H5N1 since 2011 infecting the same hosts. Subsequently, H9N2 has established an endemic status in the poultry sector. Human infections with both H7N9 and H10N8 viruses highlighted that H9N2 has an emerging state of new human infecting virus. ^16^

In Indonesia, the circulation of the H9N2 and H5N1 viruses and the possibility of reassortment between the two viruses have resulted in various virus control situations. ^17^ The LP avian influenza subtype H9N2 virus raises a public health risk. It has human-like receptor specificity ^2^^,^^18^^,^^19^ that might surpass the species barrier. ^20^^,^^21^

In 1999, LPAIV subtype H9N2 was first discovered from a human patient in Hong Kong. ^22^^,^^23^ This discovery raises concerns about the H9N2 pandemic potential alongside the H5N1 virus. ^24^^,^^25^ The recent isolation of AI H9N2 from a patient in Bangladesh and poultry workers in China has heightened public health concerns about LP avian influenza. ^26^^–^^28^ Bangladesh, Pakistan, and Egypt have all reported further cases. ^29^^–^^31^ Even though low pathogenic avian influenza H9N2 viruses could harm humans, the significance of low pathogenic H9N2 viruses has been surpassed by HPAI H5N1 viruses. ^32^ A further indication of the significance of the H9N2 subtype of the low pathogenic avian influenza virus is discovering of two other subtypes (H10N8 and H7N9) with internal genomes comparable to those of H9N2. ^33^ In the Western Pacific Region, 72 cases of avian influenza A(H9N2) infection have been reported to WHO since December 2015, including two deaths (both due to underlying diseases). ^34^

Oil-based inactivated H9N2 LPAI vaccines were used in the poultry sector in many countries to avoid H9N2 infection owing to the extensive essence of H9N2 viruses and their zoonotic potential. ^7^^,^^35^^–^^37^ However, because the nature of HA antigenic epitopes is constantly changing, influenza vaccines must be updated each year to make sure strain-specific immunity, posing a significant challenging task to vaccine manufacturers. As a result, a global flu vaccine with broad protection against conserved influenza protein regions is required.

In the Indonesian poultry industry, this review addresses critical issues concerning to the evolution of AI viruses and vaccination strategy. Vaccination against the LPAI H9N2 virus is also discussed, including recent advances and challenges.

## The emergence and evolution of the LPAI H9N2 virus

### Brief history of avian influenza in Indonesia

Till to date, poultry industry in Indonesia has faced a serious threat from highly pathogenic avian influenza (HPAI). The H5N1 virus has rapidly spread across most provinces since its initial report in 2003–2004, eventually subsiding by the end of 2007 after killing over 16 million chickens. ^38^^,^^39^ A second epidemic was recorded in Gorontalo in April 2011, leaving only one province disease-free. ^40^ A phylogenetic assessment of the Indonesian 2.1. clade virus revealed a direct relationship to viruses of genotype Z discovered in Hunan Province, China, in 2002, indicating that they were likely introduced together. However, the propagation and transmission of the virus from Hunan to Indonesia remained unknown. ^41^^,^^42^ All Indonesian H5N1 viruses were categorized as clade 2.1 up until 2008, with three virus sub-lineages: 2.1.1, 2.1.2, and 2.1.3. During the outbreaks between 2003 and 2005, the viruses of clade 2.1.1 were mostly isolated from HPAI-infected poultry. Clade 2.1.2 viruses with avian and human origins were primarily detected in Sumatra between 2004 and 2007, whereas clade 2.1.3 viruses were detected in 2004 and isolated from either birds or humans. Surprisingly, when clade 2.1.3 viruses became more prevalent, the number of clade 2.1.1 and 2.1.2 isolates began to fall. Despite the fact that 2.1.3 viruses have expanded throughout Indonesia and grown endemic in several areas, a new sub-lineage virus has arisen since 2004. In September 2012, AIV H5 subtype mortality was detected at several duck farms in Central Java. The HA genes of the duck isolates did not match those of long-established Indonesian clade 2.1 isolates, but they were surprisingly comparable to clade 2.3.2.1 viruses found lately in Vietnam, China, and Hong Kong. ^43^ Although Bali is thought to be an excellent environment for influenza re-assortment because of its world-renowned tourism destination, suckling pigs, and fighting cocks’ history, until 2017, the island had reported only one human death from avian influenza. Between 2009 and 2011, surveillance of AI (H5N1) viruses in Bali revealed that the circulating A(H5N1) viruses belonged to clade 2.1. ^44^^–^^46^

### Avian influenza subtype H9N2

In early 2017, I Ketut Diarmita, Director General of Livestock and Animal Health at the Ministry of Agriculture, Indonesia announced that newly emerging AIV subtype H9N2 was discovered during surveillance by the Ministry of Agriculture’s Veterinary Center in South Sulawesi, West Java, Bali, Central Java, and Yogyakarta. As a result of these incidents, egg supply has decreased by the end of 2017. ^47^

According to Drh. Ni Made Ria Isriyanti, Ph.D., Head of Sub-Supervision of Veterinary Medicine, Directorate General of Livestock and Animal Health, Indonesia, the current state of the H9 virus is its proliferation in several provinces in Indonesia, including Java, Sumatera, Kalimantan, Sulawesi, and Bali. The number of H9N2 positive samples amounted to 49. Infected chickens are typically 30–60 weeks old. Although mortality is normally modest, one indication of the H9 virus is a decrease in egg production of up to 40–60% of normal, resulting in significant economic losses for farmers. ^48^ The LPAI virus subtype H9N2 has been circulating in poultry and ducks in Indonesia, causing significant financial losses. It was also happening because of higher mortality and decreased production, particularly in broiler and layer chickens. ^15^ Since 2003, the HPAIV subtype H5N1 highly pathogenic avian influenza (HPAI) virus has been found in Indonesia, ^49^ with human cases resulting from H5N1 viruses being transmitted cross-species.

A study conducted by Muflihanah *et al.* (2017) in Sidrap Regency, South Sulawesi found that the occurrence of AIV disease occurs within a period of 3–14 days, with an average mortality rate of less than 5% and a 50–80 percent decline in egg production. The genetic similarity of three isolates A/Chicken/Sidrap/07161511-1/2016, A/Chicken/Sidrap/07161511-61/2016, and A/Chicken/Sidrap/07170094-44OA/2017 is 98 percent H9N2. The phylogenetic tree results suggest that the tested sample appears to be from the Asian group or lineage Y280-H9N2. ^50^

Another study conducted by Nugroho *et al.* (2018) in layer chicken in Java Island, 13 of the 33 virus isolates were VAI subtype H9N2 and belonged to the Y280 lineage, clade h9.4.2.5, and had genetic closeness with Chinese isolates in 2013 and Vietnam in 2014, with a nucleotide homology percentage of 96.9 percent–98.8 percent. ^51^

According to a study conducted by Wibawa H, *et al.* (2020) phylogenetic analysis of H9N2 virus HA9 gene (Bt/1291-OP/16) was found to be part of the China-Vietnam-Indonesia linage (CVI lineage). ^52^

**Figure 2.**
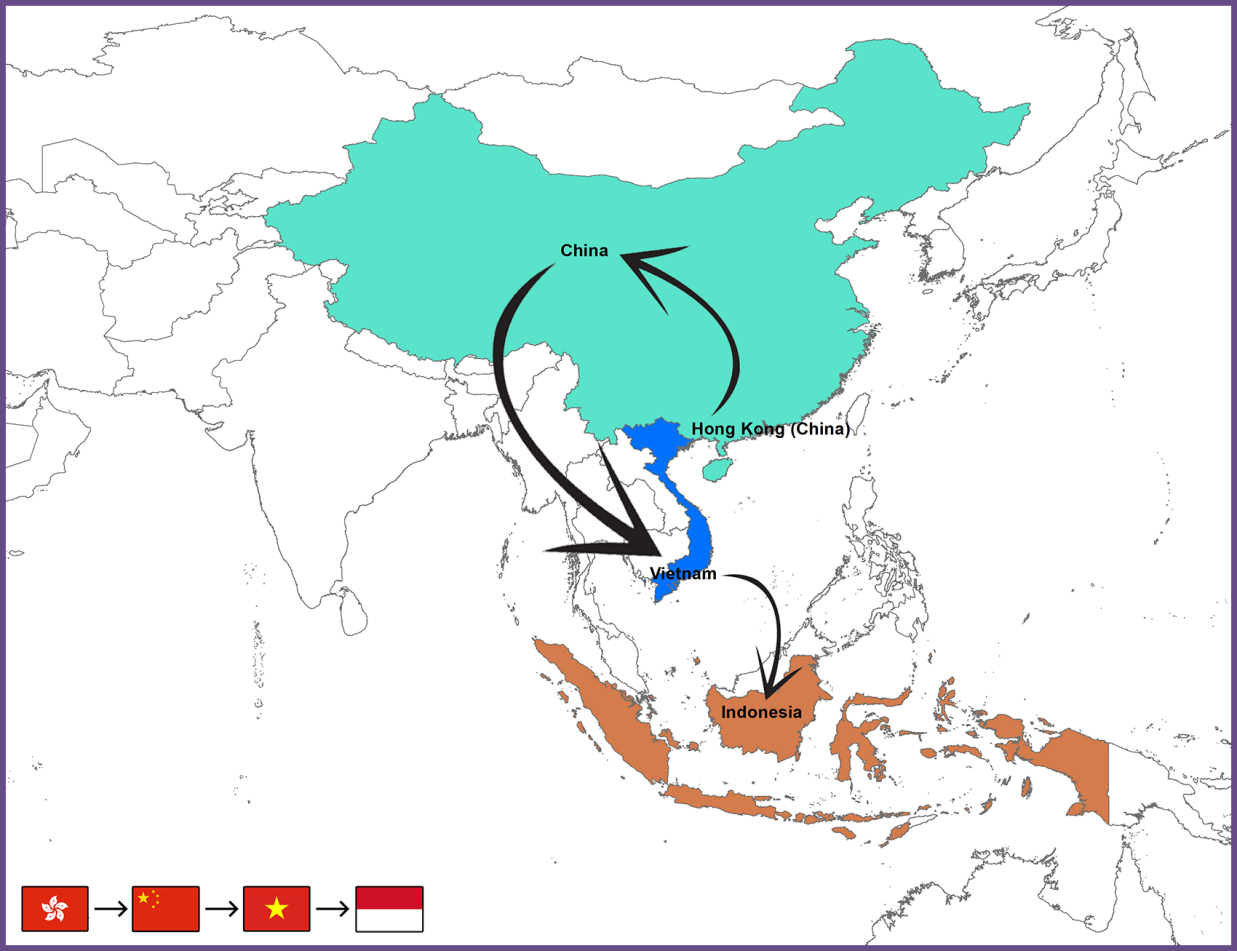
Map depicts the proposed and hypothesized pathways of the avian influenza H9N2 virus taken from Hong Kong to Indonesia.

It indicated a close relationship with H9N2 viruses prevalent in China and Vietnam. That is why it was classified with the H9N2 viruses of the China Vietnam-Indonesia (CVI) lineage. Vietnam H9N2 viruses (H7F-LC4-51/14, H7F-LC4-26/14, and H7F 14 BN4 423/14) had already been recognized as members of the Y280-like group. ^53^ The probable transmission paths of the AIV subtype H9N2 from Hong Kong to Indonesia ( Figure 2).

**Figure 3.**
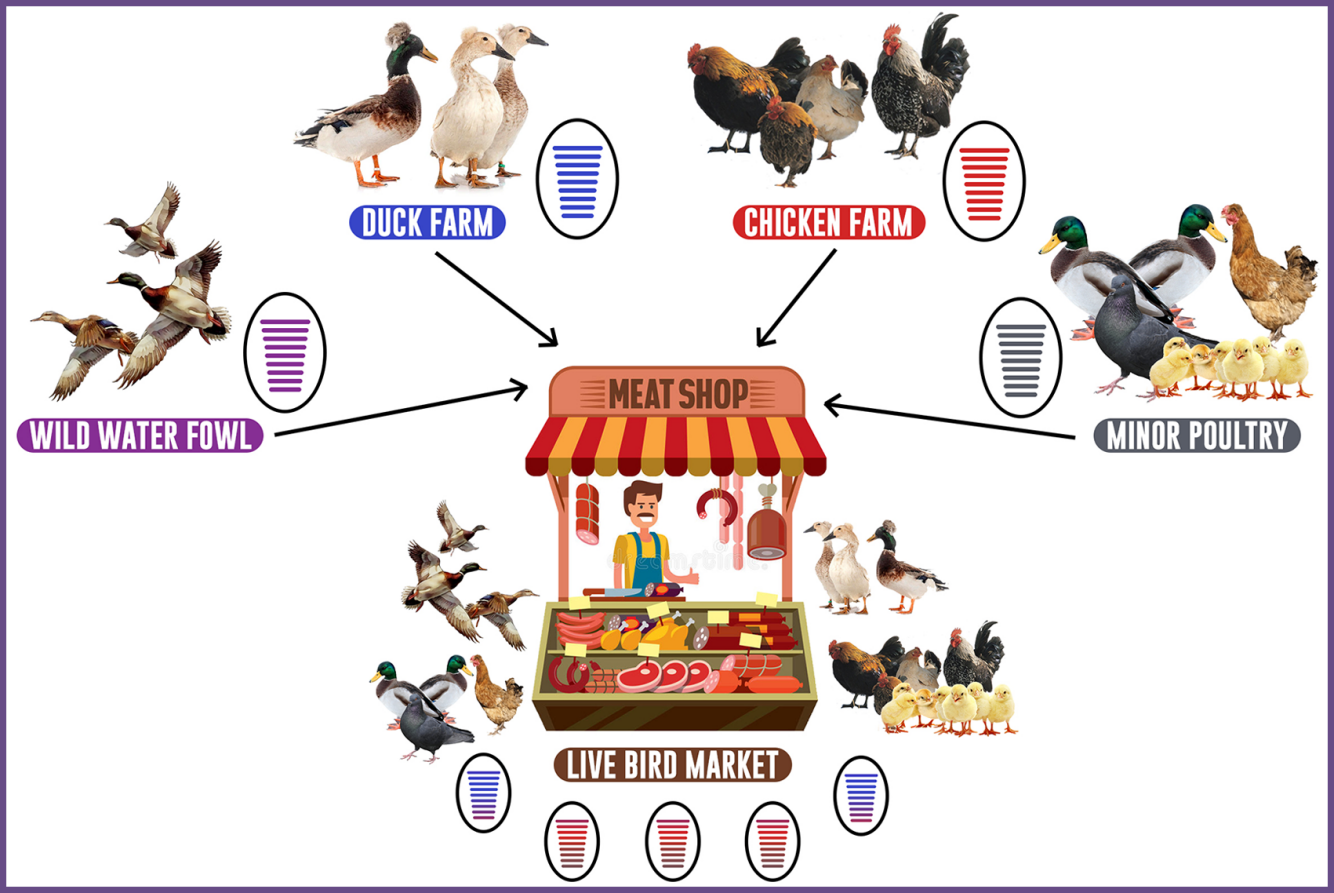
Role of live bird markets assisting in the evolution of LPAI virus H9N2 in Indonesia.

Live and Wet bird markets play an essential role in the ecology of HPAI subtype H5N1 and LPAI subtype H9N2 in Indonesia ( Figure 3) and are a critical factor in the disease’s prevalence and endemicity. ^54^ The co-circulation of H5N1 and H9N2 viruses in poultry farming and live bird markets have raised the danger of human infection, complicating the epidemiological picture and heightening fears of a new influenza A virus pandemic. ^55^

In Indonesia, primarily poultry (layer, backyard, and broiler) and duck are raised in conventional methods on small outdoor farms with poor management, and are primarily sold through Wet and LBMs. Ducks, commercial and domestic poultry, pigeons, starlings, quails, and other species of fancy birds are among the avian species found in Wet and LBMs. ^56^^,^^57^

Some farmers have begun to grow chickens and ducks in semi-intensive or intense ways. In Indonesia, conventional farming involves herding ducks and poultry onto open rice fields after harvest to consume leftover rice, other grains, and insects. ^58^ H9N2 and high pathogenic avian influenza focused on continuing avian influenza surveillance. The subtype H5N1 was found in chicken farms and backyard chickens traded in LBMs. ^15^ This previously confirmed findings that the trade of poultry, ducks and other birds in live bird markets (LBMs) played a crucial role in discovering a new AIV. ^59^^,^^60^ According to a research, H9N2 viruses may operate as “new ventures” or “implementers” for human-infecting wild-bird influenza viruses (H7N9, H10N8). ^61^^,^^62^

Moreover, any reassortment of LPAI H9N2 viruses with highly pathogenic avian viruses may result in a more remarkable ability to cause human infection. ^63^ Together with the tropical temperature in this region, these features allow long-term survival, multiplication, and spread among various chicken species, as well as transfer from chicken to humans. These variables also provide enough possibilities for existing influenza viruses, such as H9N2 and H5N1, to rejoin and form newer viruses with different host specificity. In Indonesia’s wet and live bird markets, the broad co-circulation of H9N2, H7N9, and high pathogenic H5N1 acts as a perfect mixing vessel for forming novel influenza subtypes. It is making the country a hotspot for the AI epidemic. Comprehensive vaccination programs have been implemented to mitigate the effects of H5, particularly newly emerging H9 subtype viruses spreading in Indonesia. ^14^

## Prospects for AI vaccination in the future

In Indonesia, vaccination is one of the most effective ways to combat the spread of avian influenza (AI) viruses. The vaccine master seed used in the field must be updated to keep up with the variety of circulating viruses and their potential to change. A vaccination strain (LPAI H9N2) virus isolated in 2017 (A/chicken/West Java/BBLitvet-RI/2017) vaccine (Patent IDP000056903) ^64^ and BLi25Ut/18 virus were chosen in Indonesia based on their pathogenic, antigenic, and genetic features. Inactivated bivalent and monovalent H9N2 influenza vaccinations can induce an antibody response. It can lower mortality and virus shedding caused by reassortant H9N2 virus infection. ^17^ With the help of FAO/OFFLU, the Indonesian government has built an effective vaccination strategy against H5N1 and H9N2 strains. Influenza Virus Monitoring (IVM online) is a web-based animal health laboratory system. This system manages antigenic and genomic data of circulating HPAI and LPAI viruses in Indonesia. ^65^ Animal Disease Investigation Centers (DICs), private companies, and universities collaborate to monitor, collect isolates. The data is then submitted to IVM Online, which provides an up-to-date map of circulating HPAI and LPAI virus throughout Indonesia, allowing the optimal AI vaccine to be prescribed. In backyard farms, HPAI vaccines are commonly used to prevent LPAI using homologous (H5N1) or mixed with H9N2 strains.

In Indonesia, oil-based inactivated bivalents and monovalent vaccinations produce detectable antibody titers for all structural proteins, especially nucleoprotein and matrix protein. Antigens for antibody testing can be one or both of these proteins. As a result, using this method vaccinated birds cannot be discriminated from naturally sick birds. The inability to conduct surveillance has been a critical impediment to vaccination to combat avian influenza. There has been a lot of effort put towards matching the vaccination to the field variations. This is partly because immunization with any H9 virus appears to protect against clinical illness from a low pathogenic avian influenza exposure of the same subtype, irrespective of genetic variations. Oil-based inactivated bivalent and monovalent vaccinations produce many serum antibodies. The heterogeneity between vaccine and field strain can be estimated by comparing genomic information in the HA gene. However, when the vaccine is utilized as a control tool, both clinical safety and virus replication are concerns. According to experimental research, the closer the vaccine is to the field strain, the less virus is released in exposed birds. ^37^ Genetic variation is a significant issue with avian influenza vaccines, as it reduces immunization effectiveness. Antigenic drift is considered to unfold when the field virus changes in response to the host’s antibodies. This method could be owing to vaccination or natural infection. However, in any scenario, the virus is under evolutionary changes to elude the body immunity, allowing multiplication at more significant titers in the host. There is a higher probability that a strain of the virus will spread to new hosts if the proliferative phase is better managed.

Virus detection has decreased in Indonesia following vaccination programs against HPAI H5N1, showing that HPAI H5N1 is now under control. While the LPAI H9N2 virus is a new subtype, recent research has shown that monovalent and bivalent vaccines can protect chickens against reassortants H9N2 virus infections. It could lower mortality and virus shedding in chickens. ^17^ Active surveillance of chicken farms and live bird markets is essential for further identifying new variants of the LPAI H9N2 virus in Indonesia. In order to prevent future epidemics, suitable vaccine seed viruses should be evaluated. Differentiation Infection in Vaccinated Animals (DIVA), a vaccine strategy, could be useful in assuring trading partners of the safety of poultry and poultry products. It has enhanced surveillance to detect virus infections. ^66^ West Java has tested a proposed DIVA technique involving sentinel chickens. ^67^^,^^68^ In Indonesia, the DIVA approach has not been widely accepted. Several different ways for employing viral protein as a marker in chickens, such as HA2, ^69^ NS1 ^70^ and M2e ^71^ have been developed. New prospects for developing novel concept vaccines arise due to better molecular virology and the accessibility of genetic data on avian influenza. VLPs (Virus-like particles) have been proposed as a new generation of non-egg-based vaccinations with potential safety profiles for some viral illnesses. ^72^^–^^74^ VLP is structurally and morphologically similar to infectious virus particles. Various antigenic epitopes have been shown to be particularly effective, owing to their ability to induce a wide spectrum of immune responses in the host. ^75^^,^^76^ Insect or mammalian cells can easily create virus-like particles (VLP) vaccines incorporating influenza hemagglutinin (HA) and neuraminidase (NA) antigens by expressing HA and NA proteins together with a viral core protein, such as influenza M1.

The majority of influenza VLPs were created using viral nucleic acid expression methods. Their safety and immunogenicity were tested in various animal models. ^76^^,^^77^ The H5N3 avian influenza virus-like particles (VLP) vaccine was studied in ducks. This study has demonstrated that the VLP vaccination may be administered safely in poultry. ^78^ In a specified pathogen-free (SPF) chicken model, a VLP vaccination including the HA and M1 proteins was designed and tested against H9N2 LPAI. ^32^^,^^35^ The pure VLP protein solution can be emulsified with Montanide ISA70 oil adjuvant (Seppic, Paris, France) to make a VLP vaccine. A single dose of H9 VLP vaccination resulted in significant antibody titers and reduced expulsion and release of virus progeny from the respiratory and gastrointestinal tracts in chickens. Furthermore, it enabled ELISA-based discrimination of avian influenza-infected poultry from vaccinated poultry utilizing a nucleocapsid antigen, availed DIVA approach. ^79^ On the other hand, vaccination cost is regarded to be a major factor influencing the efficacy of synthetic subunit vaccines, such as VLP for poultry. Two subunits make up the influenza virus’s haemagglutinin (HA). The current influenza vaccine largely produces antibodies against the HA1 component, which is continually developing unexpectedly. The other component, HA2, is more stable, but the HA head region protects it. As a result, increasing the immunological response to HA2 may elicit broadly inhibiting antibodies. ^80^^,^^81^ For the activation of protecting immune responses against infectious diseases, DNA vaccination has emerged as a viable alternative for standard protein-based vaccines. DNA vaccines have many advantages over traditional vaccinations, including greater stability, quick and low-cost manufacture, and the capacity to create vaccines for a broad range of infectious diseases. After being inoculated directly into mouse muscle for the first time in 1990, it was discovered that plasmid DNA vaccines could be made for the first time. ^81^^–^^83^ These DNA vaccines are capable of encoding a chimeric DNA molecule of numerous antigenic sequences, which decreases production time and costs when compared to the traditional vaccinations we now use, without carrying the illnesses related with live attenuated vaccines. These vaccines based on plasmids can trigger both immune responses (humoral and cellular) while expressing high amounts of proteins of interest in cells. They can also neutralize antibodies produced by the mother. ^84^^,^^85^

## Conclusion

In Indonesia, the avian influenza subtype H5N1 is still endemic. In 2017, the newly developing subtype LPAI H9N2 was reported for the first time in Indonesia, on the island of Java. According to a previous study, H9N2 viruses have experienced substantial genetic reassortment in recent years, resulting in novel genotypes of H9N2 viruses in Indonesia. H9N2 virus genotypes that have recently emerged could play a vital role in the disease’s transmission in poultry and ducks. In order to detect future evolution and potential adaption of the LPAI H9N2 virus to humans and other mammalian species, active surveillance of these viruses is required in Indonesia. The widespread use of AI vaccinations in populations of animals may raise immunological selection pressure and mutation rates, which can lead to fast antigenic drift at antigenic locations. Better vaccination procedures and regular updating of vaccine seed variants are needed to boost immunogenicity and protective efficacy on poultry and duck farms. These techniques might be involved in selecting highly immunogenic vaccine seed strains, using efficient adjuvants for chickens and ducks, and utilizing innovative technology. In Indonesia, the co-circulation of H9N2 and H5N1 viruses in the field and live bird markets will increase the chances of gene reassortment between the viruses. Continued intensive monitoring of chicken farms and live bird markets for new variant low pathogenic H9N2 viruses and investigation of relevant vaccine seed viruses should be explored for future prevention. In Indonesia, inactivated bivalent and monovalent vaccinations have been utilized, and numerous new technology vaccines have been proposed to create low-cost, high-immunogenic vaccines. Together with efficient adjuvants, these novel vaccinations will undoubtedly lead to improved immunity against low pathogenic avian influenza subtype H9N2. In Indonesia, vaccination must be included in a complete, integrated disease-control strategy. National monitoring must be maintained at all times, as well as agricultural biosecurity and the DIVA strategy. In Indonesia, the eradication of these viruses could only be accomplished if all components of the control implemented.

## Data availability

No data are associated with this article.
