# Supplementary material for: Emerging threats and vaccination strategies of H9N2 viruses in poultry in Indonesia: A review
Source: F1000Res. 2022 Jun 28;11:548. Originally published 2022 May 19. [Version 2] doi: 10.12688/f1000research.118669.2 (PMC9253659; doi:10.12688/f1000research.118669.2)
Supplement: Peer review history

# Peer review: Reviewer response for version 2

**DOI:** 10.5256/f1000research.135273.r142576

**Published:** 2022-07-04

**Authors:** Asghar Abbas

**Affiliations:** 1 Faculty of Veterinary and Animal Sciences, Muhammad Nawaz Shareef University of Agriculture, Multan, Pakistan

I have reviewed the revised version of the manuscript, come to conclusion that authors have addressed my previous minor comments, and that it contains valuable information on Avian Influenza subtype H9N2. I believe the article is acceptable for indexing in its present form. Thanks.

Is the review written in accessible language?

Yes

Are all factual statements correct and adequately supported by citations?

Yes

Are the conclusions drawn appropriate in the context of the current research literature?

Yes

Is the topic of the review discussed comprehensively in the context of the current literature?

Yes

Reviewer Expertise:

Pathobiology  Research

I confirm that I have read this submission and believe that I have an appropriate level of expertise to confirm that it is of an acceptable scientific standard.

# Peer review: Reviewer response for version 2

**DOI:** 10.5256/f1000research.135273.r142575

**Published:** 2022-07-01

**Authors:** Agumah Nnabuife Bernard

**Affiliations:** 1 Department of Applied Microbiology, Faculty of Science, Ebonyi State University, Abakaliki, Nigeria

I have no further comments to make. I affirm my earlier stance on approving the article for publication.

 Thank you

Is the review written in accessible language?

Yes

Are all factual statements correct and adequately supported by citations?

Yes

Are the conclusions drawn appropriate in the context of the current research literature?

Yes

Is the topic of the review discussed comprehensively in the context of the current literature?

Yes

Reviewer Expertise:

I am a medical Microbiologist with specialties in Fungi of both medical and veterinary importance. I am also a certified Medical Laboratory Scientist with specialties in Medical Parasitology and entomology

I confirm that I have read this submission and believe that I have an appropriate level of expertise to confirm that it is of an acceptable scientific standard.

# Peer review: Reviewer response for version 1

**DOI:** 10.5256/f1000research.130513.r138445

**Published:** 2022-06-16

**Authors:** Agumah Nnabuife Bernard

**Affiliations:** 1 Department of Applied Microbiology, Faculty of Science, Ebonyi State University, Abakaliki, Nigeria

I have gone through the review article. The author did wonderfully well as the facts and figures were aptly captured with most categorical statements backed up with appropriate citations.

 The author did well in:

1. Capturing the history and the first documentation of the H9N2 Avian Influenza in Indonesia.
2. Noting the fact that the contemporary low pathogenic nature exhibited by the ethiologic agent and its prospects of becoming highly pathogenic in the nearest future. This is also a good headway.
3. It is worthy of note that the author threw good light to the prospects of antigenic drift and zoonotic transmission.
4. Though only the Hongkong and the Korean genotypes were explained by way of history; the author was able to fully highlight how Indonesia currently has a very high distribution of H9N2 in Indonesian poultry Industry.
5. It is also worthy of note that the author highlighted how the conventional methods used in raising birds has contributed in the endemicity of the H9N2 Avian Influenza in Indonesia. Poor management and sales in wet and live birds market are well cited as contributing factors in the spread of Avian influenza.
6. The author was able to highlight various subtypes especially the H5N1 which caused the Bird flu pandemic that lasted from 2003-2007.
7. The most novel information from the authors review is that it is highly likely that the next form of Influenza to be contracted by humans in Indonesia is the H9N2 Avian influenza. Hence adequate surveillance and analysis of the genetic flexibility of the virus opens up a huge prospect for vaccine development should the H9N2 virus infect humans.

A little correction I may suggest is with the topic. It should read Emerging threats and not threat. . . . as there may be many variables with respect to the medical importance of the H9N2 virus in Poultry in Indonesia.

Is the review written in accessible language?

Yes

Are all factual statements correct and adequately supported by citations?

Yes

Are the conclusions drawn appropriate in the context of the current research literature?

Yes

Is the topic of the review discussed comprehensively in the context of the current literature?

Yes

Reviewer Expertise:

I am a medical Microbiologist with specialties in Fungi of both medical and veterinary importance. I am also a certified Medical Laboratory Scientist with specialties in Medical Parasitology and entomology

I confirm that I have read this submission and believe that I have an appropriate level of expertise to confirm that it is of an acceptable scientific standard.

# Response

**Published:** 2022-06-24

**Authors:** Saifur Rehman

**Affiliations:** Airlangga University, Indonesia

**2 ^nd^ Reviewer comments**

1. A minor correction I may suggest is with the topic. It should read Emerging threats and not threat.  as there may be many variables with respect to the medical importance of the H9N2 virus in Poultry in Indonesia.
**Response: **We have changed the Title according to your suggestions as “Emerging threats and vaccination strategies of H9N2 viruses in poultry in Indonesia: A review".

# Peer review: Reviewer response for version 1

**DOI:** 10.5256/f1000research.130513.r138444

**Published:** 2022-06-08

**Authors:** Asghar Abbas

**Affiliations:** 1 Faculty of Veterinary and Animal Sciences, Muhammad Nawaz Shareef University of Agriculture, Multan, Pakistan

Dear Editor/Authors,

 I have review of the manuscript "Emerging threat and vaccination strategies of H9N2 viruses in poultry in Indonesia: A review". The Article is well written and contains valuable information on Vaccination against Avian influenza Disease. However the manuscript needs minor revisions and improvement as per following comments

1. English Language of whole manuscript should be revised and improved.
2. Justification (4-5 lines) of study should be added in introduction with proper latest references.
3. Kindly add Associated risk factors of Avian influenza disease in Indonesia.

I recommend manuscript be accept after minor revision.

Is the review written in accessible language?

Yes

Are all factual statements correct and adequately supported by citations?

Yes

Are the conclusions drawn appropriate in the context of the current research literature?

Yes

Is the topic of the review discussed comprehensively in the context of the current literature?

Yes

Reviewer Expertise:

Pathobiology  Research

I confirm that I have read this submission and believe that I have an appropriate level of expertise to confirm that it is of an acceptable scientific standard, however I have significant reservations, as outlined above.

# Response

**Published:** 2022-06-24

**Authors:** Saifur Rehman

**Affiliations:** Airlangga University, Indonesia

Dear Reviewer, 

 Thanks for your valuable comments and suggestions on the manuscript entitled:

 “ **Emerging threats and vaccination strategies of H9N2 viruses in poultry in Indonesia: A review”**

 We welcome feedback. We have made modifications to the study on the following points:

- English Language of the whole manuscript should be revised and improved
**Response:** We have revised the English language of the whole manuscript.
- Justification (4-5 lines) of study should be added in the introduction with proper latest references.
**Response:** We have added the Justification of the study in the Introduction section.
- Kindly add the Associated risk factors of Avian influenza disease in Indonesia.
**Response:** We have added the associated risk factors of AIVs which showed association with AIV in Indonesia
